# A Novel Electrochemical Microfluidic Chip Combined with Multiple Biomarkers for Early Diagnosis of Gastric Cancer

**DOI:** 10.1186/s11671-015-1153-3

**Published:** 2015-12-10

**Authors:** Yao Xie, Xiao Zhi, Haichuan Su, Kan Wang, Zhen Yan, Nongyue He, Jingpu Zhang, Di Chen, Daxiang Cui

**Affiliations:** Department of Instrument Science and Engineering, Institute of Nano Biomedicine and Engineering, Key Lab. for Thin Film and Microfabrication Technology of Ministry of Education, School of Electronic Information and Electrical Engineering, Shanghai Jiao Tong University, 800 Dongchuan Road, Shanghai, 200240 Peoples’ Republic of China; Department of Oncology, Tangdu Hospital, Fourth Military Medical University, 569 Xinsi Road, Xi’an, 710032 Peoples’ Republic of China; Department of Pharmaceutics, Fourth Military Medical University, 18 Changle West Road, Xi’an, 710032 Peoples’ Republic of China; State Key Laboratory of Bioelectronics, School of Biological Science and Medical Engineering, Southeast University, Nanjing, 210096 Peoples’ Republic of China; Institute of Translation Medicine, Tumor Personalized Therapy and Molecular Diagnosis Base of Ministry of Health and Family Planning Commission, Collaborative Innovational Center for System Biology, Shanghai Jiao Tong University, 800 Dongchuan Road, Shanghai, 200240 Peoples’ Republic of China

**Keywords:** Electrochemical, Microfluidic chip, Multiple biomarkers, Early diagnosis, Gastric cancer

## Abstract

Early diagnosis is very important to improve the survival rate of patients with gastric cancer and to understand the biology of cancer. In order to meet the clinical demands for early diagnosis of gastric cancer, we developed a disposable easy-to-use electrochemical microfluidic chip combined with multiple antibodies against six kinds of biomarkers (carcinoembryonic antigen (CEA), carbohydrate antigen 19-9 (CA19-9), *Helicobacter pylori* CagA protein (H.P.), P53oncoprotein (P53), pepsinogen I (PG I), and PG-II). The six kinds of biomarkers related to gastric cancer can be detected sensitively and synchronously in a short time. The specially designed three electrodes system enables cross-contamination to be avoided effectively. The linear ranges of detection of the electrochemical microfluidic chip were as follows: 0.37–90 ng mL^−1^ for CEA, 10.75–172 U mL^−1^ for CA19-9, 10–160 U L^−1^ for H.P., 35–560 ng mL^−1^ for P53, 37.5–600 ng mL^−1^ for PG I, and 2.5–80 ng mL^−1^for PG II. This method owns better sensitivity compared with enzyme-linked immunosorbent assay (ELISA) results of 394 specimens of gastric cancer sera. Furthermore, we established a multi-index prediction model based on the six kinds of biomarkers for predicting risk of gastric cancer. In conclusion, the electrochemical microfluidic chip for detecting multiple biomarkers has great potential in applications such as early screening of gastric cancer patients, and therapeutic evaluation, and real-time dynamic monitoring the progress of gastric cancer in near future.

## Background

Gastric cancer (GC) is the second most common cancer and the third leading cause of cancer-related death in China [1–3]. It remains very difficult to cure effectively, primarily because most patients present with advanced diseases. Up to date, gastric cancer prognosis is very poor with 5-year survivals below 24 %. Multidisciplinary treatment is used to improve treatment efficacy of advanced stage of GC. However, it has been proven that gastric cancer is not particularly sensitive to current chemotherapy agents, which is closely associated with intrinsic or acquired properties of gastric cancer cells. Therefore, discovery of early gastric cancer has become main pathway to improve the therapeutic efficacy.

We have tried to establish an early gastric cancer pre-warning and diagnosis system since 2005 [4]. We hoped to find early gastric cancer cells in vivo by multi-mode targeting imaging and serum biomarker detection techniques. Our previous studies showed that subcutaneous and in situ gastric cancer tissues with 5 mm in diameter could be recognized and treated by using multifunctional nanoprobes such as breast cancer-associated antigen 1(BRCAA1)-conjugated fluorescent magnetic nanoparticles [5], human epidermal growth factor receptor-2 (HER-2) antibody-conjugated Rnase A (ribonuclease A)-associated CdTe quantum dots [6], folic acid-conjugated upper conversion nanoparticles [7], Arg-Gly-Asp (RGD) peptide-conjugated gold nanorods [8], ce6-conjugated carbon dots [9], ce6-conjugated Au nanoclusters (AuNCs) [10], HAI-178 antibody-conjugated fluorescent magnetic nanoparticles [11], CD44 monoclonal antibody-conjugated gold nanostars [12], and RNA nanoparticles carrying both ligand and siRNA [13]. However, clinical translation of these prepared nanoprobes still exist great challenge because their biosafety still needs a long term evaluation. We also screened out some breath biomarkers associated with gastric cancer [14], and established some methods and devices to detect these biomarkers [15–17]. We also developed a giant magneto resistive (GMR) microfluidic system to detect the circulation gastric cancer cells [18]. However, up to date, serum biomarker detection to screen or find early gastric cancer is still most effective method.

In the past decades, detection of serum tumor biomarkers has always been an important mean of diagnosis of various cancers. However, accepted unique serological biomarker for gastric cancer, like as Alpha-fetoprotein (AFP) for hepatocellular carcinoma (HCC) [19], remains absent. For this reason, combined detection of multiple serological biomarkers is an alternative effective method for predicting risk of gastric cancer. Several serological biomarkers based on a lot of literature can be used for early diagnosis of gastric cancer so far. Mutations in the tumor suppressor gene *p53* are the most commonly observed in human cancers. In the serum of healthy subjects, the presence of P53 protein is extremely rare. Mutations in this gene cause an accumulation of nonfunctional proteins. The accumulated proteins are detectable in tissues, sloughed cells, blood, and other body fluids [20]. The *p53* gene mutations are significantly correlated with P53 protein over-expression and contribute to genetic predisposition in gastric cancer patients [21–23]. Carcinoembryonic antigen (CEA) is an acknowledged member of immunoglobulin superfamily, with a role as an intracellular adhesion molecule [24]. A high-serum CEA is associated with a number of malignancies, including colorectal, breast, gastric, and pancreatic cancers [25]. CA19-9 has a positive correlation with depth of invasion, nodal involvement, and peritoneal metastasis in gastric adenocarcinoma [26, 27]. In addition, many studies have shown that serum pepsinogen I (PG I) [28, 29], pepsinogen II (PG II) [30], PG I/PG II ratio [31, 32], and *Helicobacter pylori* (H. P.) [33–35] are also associated with an increased risk of gastric cancer. So, combined detection of above serum biomarkers is helpful to enhance accuracy of predicting gastric cancer risk.

Enzyme-linked immunosorbent assay (ELISA) is widely used for clinical cancer diagnosis; nevertheless, these ordinary ELISA kits for single biomarker are not suitable for individual diagnosis, especially for patients with risk of gastric cancer. Moreover, the ELISA kits for batch samples from the different patients not only easily expose to cross-contamination, but also the operation is complicated. Self-assembled monolayers (SAMs) are widely used to immobilize biomolecules on gold surfaces [36]. The self-assembly process is the spontaneous organization of substances into gold surfaces. SAMs of different substances have frequently utilized for development of biosensors, microarrays, biochips, and molecular switches [37]. Microfluidic technology seeks to improve analysis time, decreasing the consumption of sample and reagents, diminishing the risk of contamination, consuming less power, and sensitivity through automation, integrating multiplexing analysis, and especially portability to provide the possibility of point-of-care applications [38–40]. In comparison with the methods based on chemiluminescence, fluorescence, electrochemiluminescence, or quartz crystal microbalance, electrochemical immunoassay has attracted tremendous interest due to its high sensitivity, low cost, simple instrumentation, and good portability [41]. All the same, this electrochemical immunoassay still have complicated preparation processes, high cost bring about difficult to clinical application and poor universality.

In this study, in order to meet the clinical demands and to overcome the above disadvantages, we develop a disposable easy-to-use electrochemical microfluidic chip combined with multiple antibodies for early diagnosis of gastric cancer. Optimized design of three electrodes system can effectively avoid cross disturbance. And combined detection based on multiple antibodies can improve the early diagnostic rate of gastric cancer. Accordingly, the unique electrochemical microfluidic chip owns great potential in application for gastric cancer early screening in near future.

## Methods

### Fabrication of Electrochemical Microfluidic Chip

**Fig. 1:**
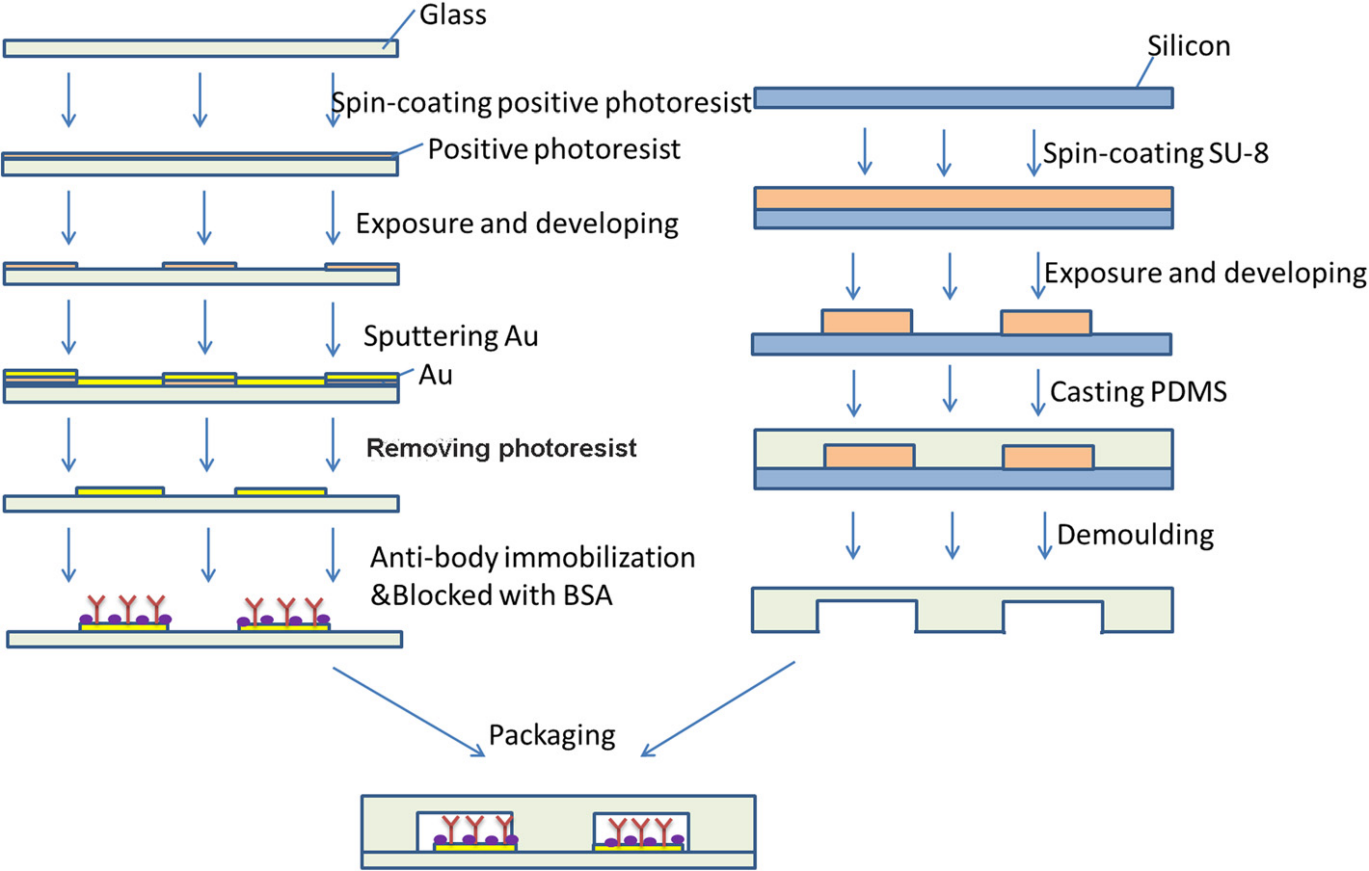
Overview of the electrochemical microfluidic chip fabrication process

Microelectrodes were fabricated on a glass wafer using standard micro-fabrication techniques. Chromium (Cr 100 nm)/gold (Au 200 nm) film stack was deposited on the glass wafers using electron-beam evaporator (L-H Inc.). Cr layer acts as the adhesion promoter for the gold film. The Au microelectrodes were formed on a glass wafer using a lift-off process as follows: a photoresist (AZ4903) was spin coated onto a glass wafer and then patterned by photolithography. Next, Au/Cr (200 nm/100 nm) was deposited onto the patterned glass wafer by electron-beam evaporator. After that, the electrodes on the glass substrate were completed by removing the photoresist from underneath the deposited metal using a solvent. Lift off was performed via sonication in acetone followed by rinsing in deionized water. Individual chips were cut using cutting machine (K&S Inc.). Each of the chips included six groups of electrodes. One group of detection electrode was comprised of working electrode, pseudo reference, and counter electrode. The surfaces of the Au electrodes were immobilized with antibodies by chemical process according to the following section of methods. PDMS (polydimethylsiloxane) molds were fabricated by photolithography of SU-8 photoresist on Si wafers and the thickness of SU-8 is 30 μm. PDMS pre-polymer and curing agents were mixed, degassed and poured onto the molds, and cured at 60 °C for 3 h. Individual PDMS chips were cut and inlet/outlet holes were punched. Briefly, PDMS surfaces were exposed to oxygen plasma (DQJ-150, Shanghai, China). PDMS channels were assembled together with glass chips Fig. 1.

### Antibodies Immobilization

Firstly, the electrodes were washed ultrasonically in ethanol for 5 min and then were immersed in piranha solution (H_2_O_2_(v)/H_2_SO_4_(v) = 1/3) for 5 min to clean the surfaces. Subsequently, the electrodes were rinsed with sterile ultrapure water for 10 times and were dried with nitrogen. After dripping 2 μL mercaptoacetic acid (Sigma, USA) on the surface of each working electrode, the chip was placed in an airtight container for 1 h to form a carboxylic self-assembled monolayer. Then, the chip was rinsed with ethanol and was dried with nitrogen gently. The carboxyl groups on the surface of electrodes were activated with 0.4 M 1-ethyl-3-[3-dimethylaminopropyl] carbodiimide hydrochloride (EDC) and 0.2 M N-hydroxysuccinimide (NHS) solution prepared in a 0.1 M phosphate buffer solution (PBS, pH = 7.4) for 20 min for immobilizing antibodies. After rinsing with PBS buffer and drying with nitrogen, six kinds of antibodies (anti-CEA, anti-CA19-9, anti-H.P., anti-P53, anti-PG I, and anti-PG II) solutions were respectively dripped on the surfaces of six working electrodes and incubated at 37 °C for 3 h. Lastly, the immunological chip was obtained after incubating 0.5 % BSA (bovine serum albumin) at 37 °C for 1 h to block non-specific binding sites and rinsing with 0.01 M PBS buffer. The prepared chips were stored at 4 °C for next immunoreaction and electrochemical detection.

### Immunological Reaction and Electrochemical Signal Detection

The immunological reaction was carried out as follows: Firstly, the six kinds of biomarkers related to gastric cancer (CEA, CA19-9, H.P., P53, PG I, and PG II) solutions were respectively prepared in a series of concentration with phosphate buffer solution (PBS, pH = 7.4) (Table. 1). Secondly, the biomarker solutions with series of concentrations were injected into the microfluidic chips and incubated at 37 °C for 30 min to form the antigen–antibody immune complexes [42–44]. In order to remove unbound biomarkers, PBS was subsequently injected into microfluidic chip for rinsing microchannel and microchamber at 100 μL min^−1^ for 5 min.

**Table 1:** The different concentration of biomarkers in PBS

Biomarkers	Concentration gradient					
CEA (ng mL^−1^)	0.37	1.11	3.33	10	30	90
CA19-9 (U mL^−1^)	10.75	21.5	43	86	172	
H.P. (U L^−1^)	5	10	20	40	80	160
P53 (pg mL^−1^)	35	70	140	280	560	
PG I (ng mL^−1^)	37.5	75	150	300	600	
PG II (ng mL^−1^)	2.5	5	10	20	40	80

After immunological reaction, antigen–antibody complexes formed on the surface of working electrodes were simultaneously detected by differential pulse voltammetry (DPV) with an electrochemical analyzer (CHI-1030, Chenhua, China). All electrochemical measurements were performed in 5 mM K_3_[Fe(CN)_6_]/K_4_[Fe(CN)_6_]-PBS (pH = 7.4) solution from −0.3 to 0.6 V at scan rate 50 mV s^−1^.

### Comparing with ELISA and Establishing Multi-index Prediction Model

Three hundred ninety-four specimens of gastric cancer sera were collected from Tangdu Hospital, East-southern Hospital affiliated to East-southern University, Xi’an Central Hospital. Gastric cancer patients were identified by pathological doctor. ELISA kits (Boster, Wuhan, China) for CEA, CA19-9, H.P., PG I, PG II, and P53 were purchased from Shanghai Reagent Company, all ELISA kits obtained the permission certification by China Food and Drug Administration (CFDA). All specimens were examined by using ELISA method and electrochemical method in Department of Clinical Biochemistry, No. 1 People Hospital affiliated to Shanghai Jiao Tong University.

### Statistical Analysis

All results are reported as means ± SD. And all data were analyzed by statistical methods including correlation and *F* test with *SPSS* 22.0 (IBM Corp., USA). The level of *P* < 0.05 was regarded as significant.

## Results and Discussion

### Electrochemical Microfluidic Chip

**Fig. 2:**
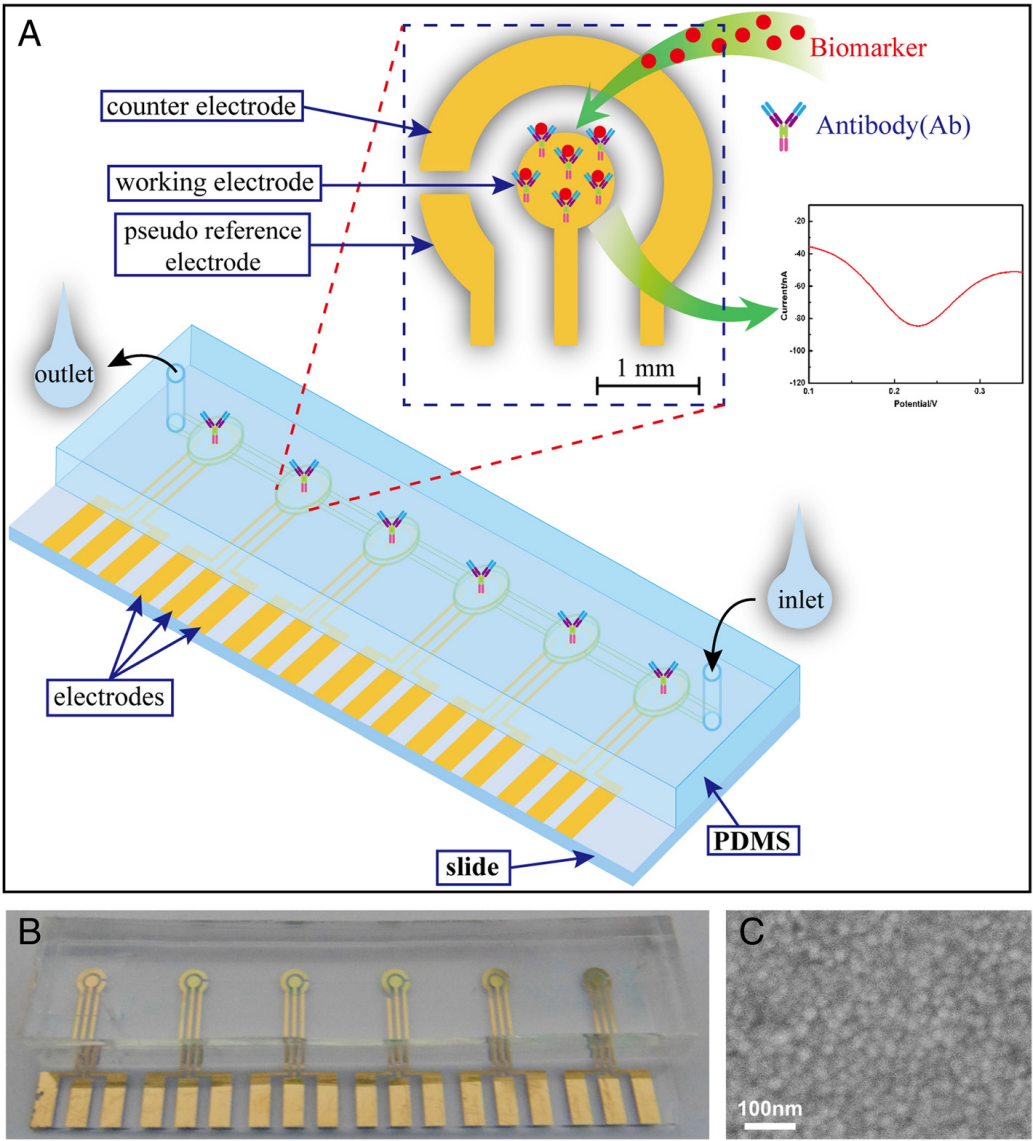
**a** Schematic illustration of the electrochemical microfluidic chip. **b** Picture of electrochemical microfluidic chip. **c** SEM image of surface of work electrode

As shown in Fig. 2b, electrochemical microfluidic chips (ECMC) were fabricated successfully. It consists of six detection areas for six different kinds of biomarkers. Every detection area was an independent gold three-electrode unit including working electrode, pseudo reference, and counter microelectrodes. As shown in Fig. 2c, the surface of electrode formed a nano-film consist of Au nanoparticles. The size of a nanoparticle is 50 ± 2 nm. The volume of single chamber is about 0.2–0.25 μL. Six kinds of antibodies against CEA, CA19-9, H.P., PG I, PG II, and P53 were respectively immobilized on surfaces of working electrodes by chemical coupling (Fig. 2a).

### Experimental Results of Six Kinds of Biomarkers

**Fig. 3:**
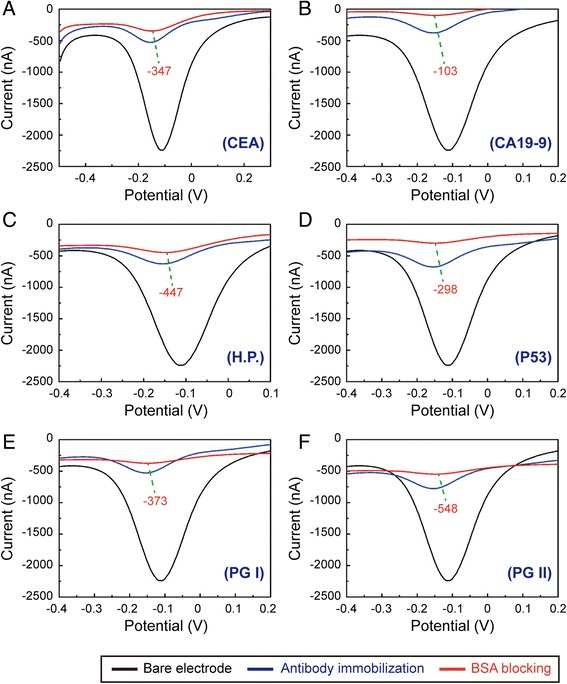
Typical differential pulse voltammograms of the electrode modified by antibodies: **a** CEA, **b** CA19-9, **c** H.P., **d** P53, **e** PG I, and **f** PG II

The electrodes of chips were characterized by differential pulse voltammetry (DPV). As shown in Fig. 3, the electrons transfer ability of working electrodes weakened obviously compared with bare electrodes (black line) after antibodies immobilization (blue line). After BSA blocking at 37 °C for 1 h (red line), the electrons transfer ability further weakened, but a little. In this study, the DPA signals of electrodes modified with antibodies after BSA blocking were defined as control signals. The control signals of working electrodes for detecting six kinds of biomarkers were −347 nA for CEA, −103 nA for CA19-9, −447 nA for H.P., −298 nA for P53, −373 nA for PG I, and −548 nA for PG II, respectively. All detectable signals stronger than the corresponding control signals were defined as positive signals.

**Fig. 4:**
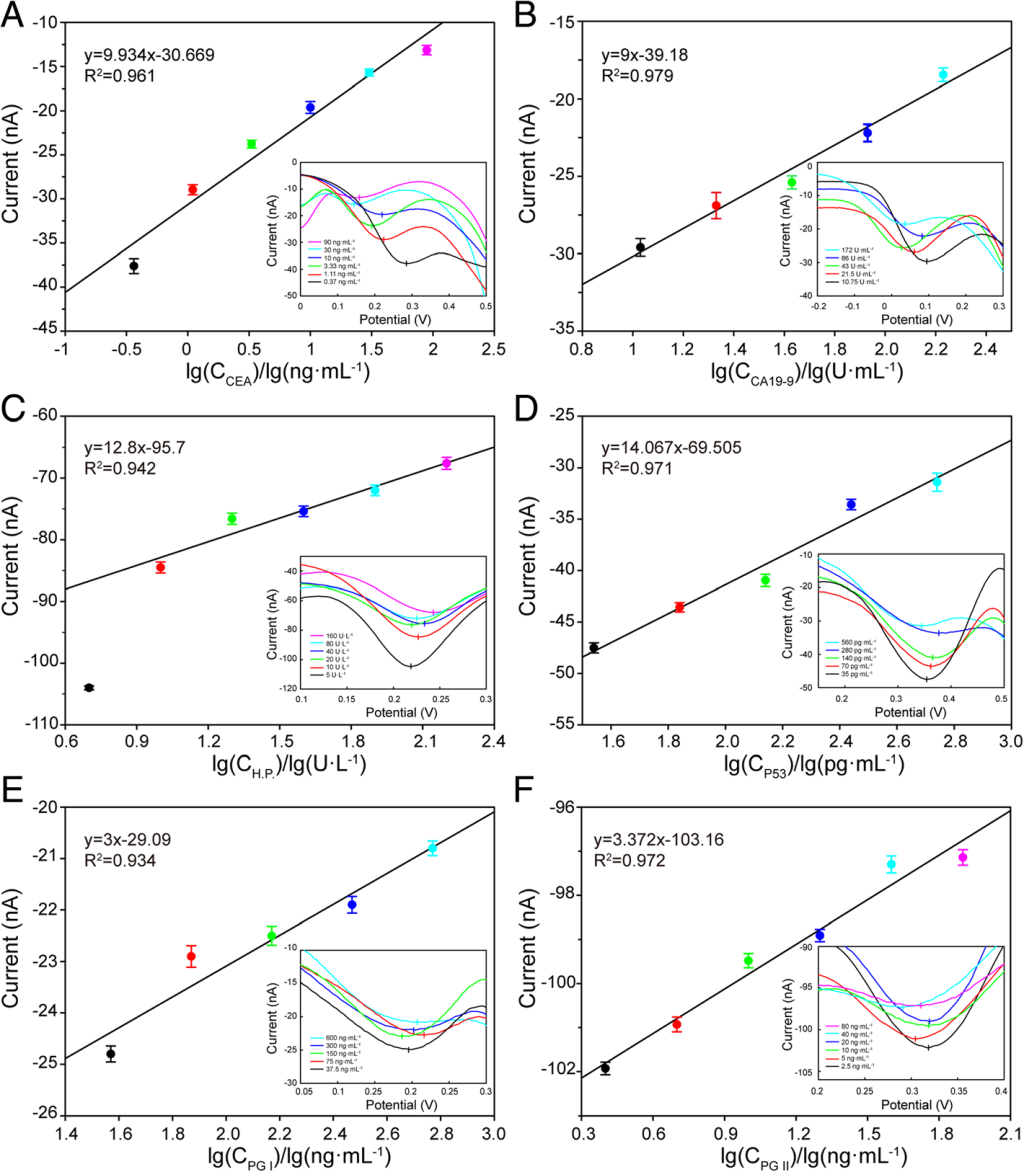
Linear detection ranges of six kinds of biomarkers by differential pulse voltammetry. **a** CEA, **b** CA19-9, **c** H.P., **d** P53, **e** PG I, and **f** PG II

The six kinds of biomarkers solutions were respectively injected into the microchannels and incubated at 37 °C for 30 min. DPV responses of K_3_[Fe(CN)_6_]/K_4_[Fe(CN)_6_] PBS were measured by multiplex electrochemical work station. As shown in Fig. 4, the oxidation currents of Fe^2+^ decreased with the biomarkers concentrations increased, and there was a positive correlation between the oxidation peak currents and the concentration of analytes. As shown in Table 2, the linear detection ranges were 0.37–90 ng mL^−1^ for CEA, 10.75–172 U mL^−1^ for CA19-9, 10–160 U L^−1^ for H.P., 35–560 ng mL^−1^ for P53, 37.5–600 ng mL^−1^ for PG I, and 2.5–80 ng mL^−1^ for PG II, respectively. And the corresponding correlation coefficients were 0.961, 0.983, 0.942, 0.971, 0.934, and 0.972, respectively. The detection limits, the minimum detectable signal higher than control signals, are 0.37 ng mL^−1^ for CEA, 10.75 U mL^−1^ for CA19-9, 5 U L^−1^ for H.P., 35 pg mL^−1^ for P53, 37.5 ng mL^−1^ for PG I, and 2.5 ng mL^−1^ for PG II, respectively. Although the detection point (5 U L^−1^ for H.P.) was not in the linear range, it still can generate a detectable response signal (Fig. 4c).

**Table 2:** The linear detection range and detection limits of biomarkers

Biomarkers	Detection range	Detection limit
CEA (ng mL^−1^)	0.37–90	0.37
CA19-9 (U mL^−1^)	10.75–172	10.75
H.P. (U L^−1^)	10–160	5
P53 (pg mL^−1^)	35–560	35
PG I (ng mL^−1^)	37.5–600	37.5
PG II (ng mL^−1^)	2.5–80	2.5

As shown in the Fig. 4, electrochemical signals of electrodes weakened owing to formation of antibody–antigen immune complexes. The immune complexed impeded of electrons transporting between the surface of electrodes and K_3_[Fe(CN)_6_]/K_4_[Fe(CN)_6_]-PBS solution. In addition, external disturbance can efficiently be avoided by independent three microelectrodes system and closed micro-chambers that are conducive to the stable electrochemical signal acquisition.

The currently electrochemical immunoassay is focused on biomarkers immobilized on nanomaterials for enhancing intensity and specificity of signal [45]. The traditional electrochemical immunoassay relied on electroactive materials and enzyme, such as HRP (horseradish peroxidase), thionine, prussian blue, and so on [46, 47]. These reported methods have inevitable limitations such as specific materials and are difficult to acquire stable signals in clinical application. At present, most of multiplex detection always detect two or three biomarkers [46, 48, 49], which cannot meet the clinical needs. This work has adopted a novel solution to avoid the above trouble. We immobilized six kinds of antibodies on the surface of microelectrodes separated by micro-chambers, respectively. An electrochemical microfluidic chip has six detection areas, each of detection area included a working electrode, a pseudo reference, a counter electrode, and a micro-chamber. Independent three electrodes system in micro-chambers avoided the crosstalk between the working electrodes. And this chip can directly detect biomarkers captured by antibodies on the surface of microelectrodes and generate response signals for early diagnosis of gastric cancer.

### Comparison with ELISA and Establishment of Multi-index Prediction Model

In order to evaluate the performance of ECMC in clinical application, 394 serum specimens of gastric cancer patients collected were used to measure the serum CEA, CA19-9, H.P., P53, PG I, and PG II levels. The normal cut-off values of CEA, CA19-9, P53, H.P., PG I, PG II, and PG I/PG II are 5.0 ng mL^−1^, 37 U mL^−1^, 10 U L^−1^, 150 pg mL^−1^, 70 ng mL^−1^, 11.5 ng mL^−1^, and 3.0, respectively [30, 31, 50–52]. As shown in Table 3, for electrochemical microfluidic chip (ECMC), the positive rates of CEA, CA19-9, H.P., P53, PG I, PG II were 7.11, 38.07, 68.78, 59.65, 74.11, and 76.74 %, respectively. For ELISA, the positive rates of CEA, CA19-9, H.P., P53, PG I, PG II were 4.57, 33.76, 52.03, 51.52, 66.75, and 65.74 %, respectively. It was evident that ECMC possessed higher detection sensitivity than ELISA.

**Table 3:** Detection performance comparison between ECMC and ELISA

Biomarkers	ECMC	ELISA	Normal reference ranges
CEA	28/394 (7.11 %)	18/394 (4.57 %)	≤5.0 ng mL^−1^
CA19-9	150/394 (38.07 %)	133/394 (33.76 %)	≤37 U mL^−1^
H.P.	271/394 (68.78 %)	205/394 (52.03 %)	≤10 U L^−1^
P53	235/394 (59.65 %)	203/394 (51.52 %)	≤150 pg mL^−1^
PG I	292/394 (74.11 %)	263/394 (66.75 %)	≤70 ng mL^−1^
PG II	301/394 (76.40 %)	259/394 (65.74 %)	≤11.5 ng mL^−1^

To explore the correlation between the six kinds of biomarkers and gastric cancer, we established a multi-index prediction model by multiple linear regression based on 394 serum samples of gastric cancer patients. The multi-index model based on large samples can be used to predict risk of gastric cancer more accurately and effectively in clinical screening.

$$ Y=b+{A}_{\mathrm{i}}\times {X}_{\mathrm{j}} $$

*A*_i_ (i = 1, 2, 3, 4, 5, 6), *X*_j_ (j = 1, 2, 3, 4, 5, 6) represented regression coefficient and detection value of six kinds of biomarkers, respectively (Table 4).

**Table 4:** Values of *b* and regression coefficient (*A*_i_)

*b*	*A*_1_	*A*_2_	*A*_3_	*A*_4_	*A*_5_	*A*_6_
9.24	5.12	1.15	–3.41	–2.58	4.31	6.83
E-01	E-06	E-04	E-06	E-05	E-05	E-06

*E* scientific notation

## Conclusions

In summary, a novel electrochemical immune detection system based on microfluidic chip was developed for simultaneous detecting multiple biomarkers (CEA, CA19-9, H.P., P53, PG I, and PG II) for early diagnosis of gastric cancer. The experimental construction and the DPV detection were based on the fact that the formed antibody–antigen immune complexes retarded the electron transfer tunnel of gold electrodes. Highlights of this work could be summarized as follows: (1) Simultaneous detection for multiple biomarkers has higher efficiency than single biomarker of ELISA. (2) The proposed microfluidic chip with independent reaction micro-chambers can contribute to stable detection signal and minimal false positive compared with conventional protein microchip systems. (3) Microfluidic chip has low detection cost because of low reagent consumption. (4) The ECMC has higher detection sensitivity and is very suitable for clinical diagnosis. (5) The multi-index prediction model can be used to predict risk of gastric cancer more accurately and effectively.

## Abbreviations

BSA: bovine serum albumin
CA19-9: carbohydrate antigen 19-9
CEA: carcinoembryonic antigen
DPV: differential pulse voltammetry
ECMC: electrochemical microfluidic chips
EDC: 1-ethyl-3-[3-dimethylaminopropyl] carbodiimide hydrochloride
ELISA: enzyme-linked immunosorbent assay
GC: gastric cancer
H.P: *Helicobacter pylori* CagA protein
NHS: N-hydroxysuccinimide
P53: P53 oncoprotein
PBS: phosphate buffer solution
PDMS: polydimethylsiloxane
PG I: pepsinogen I
PG II: pepsinogen II
SAMs: self-assembled monolayers
